# Supplementary figures and images for: Albumin promotes proliferation of G1 arrested serum starved hepatocellular carcinoma cells
Source: PeerJ. 2020 Mar 5;8:e8568. doi: 10.7717/peerj.8568 (PMC7060934; doi:10.7717/peerj.8568)

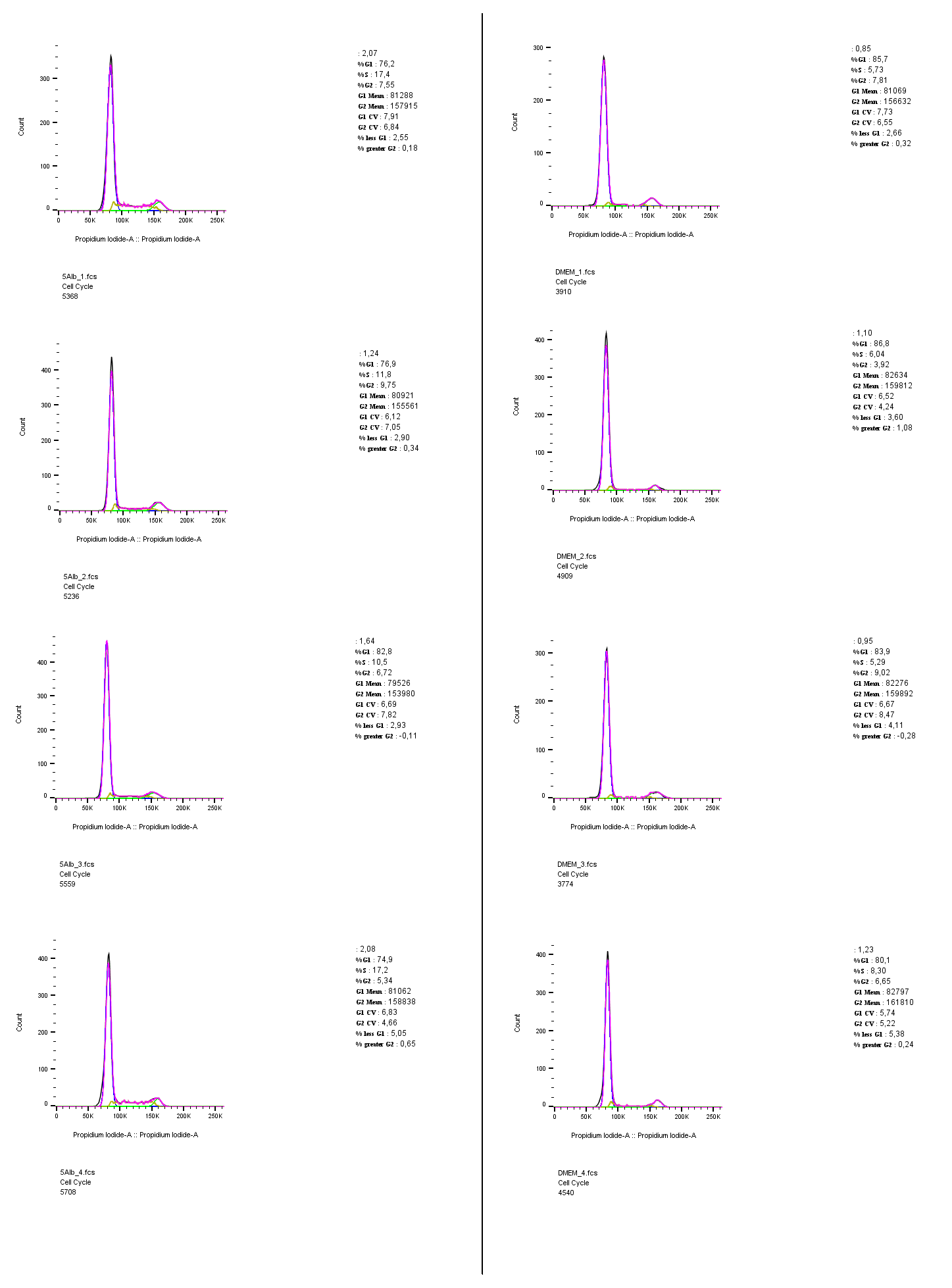

Supplement: Figure S1 — Individual histograms of the cell cycle analysis that were presented as overlays in Fig. 2. Gating, calculations and images presented in this figure were carried out using FlowJo 10.5.3. [file peerj-08-8568-s001.png]

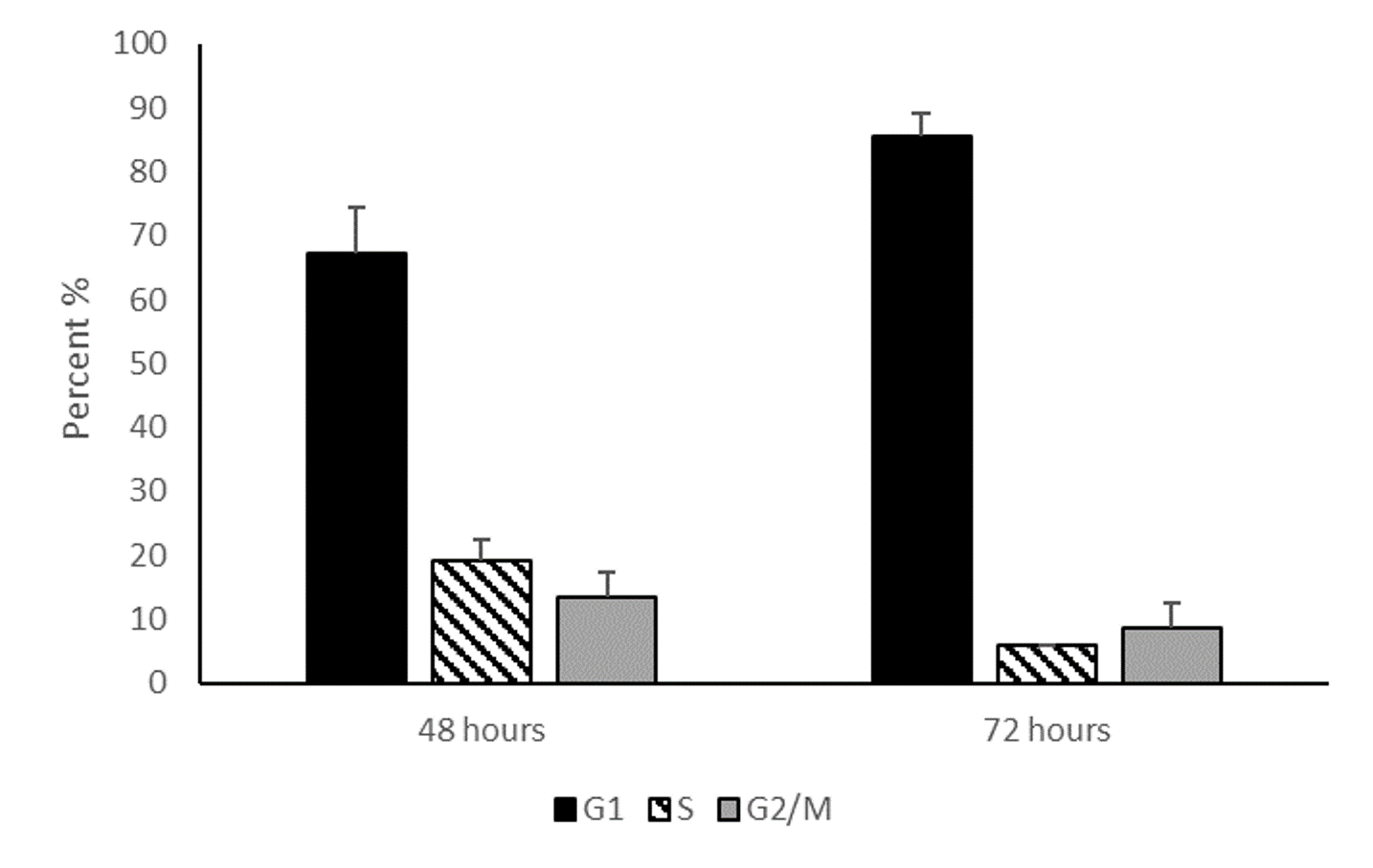

Supplement: Figure S2 — Cell cycle analysis of HEPG2/C3A hepatocytes after 48 h and 72 h serum starvation demonstrate that cell cycle arrest gradually increases over time. Data represented in the graph are mean percentages of G1, S and G2/M cell cycle stages at 48 h and 72 h of serum starvation. Values are mean ± SD (n = 2). [file peerj-08-8568-s002.png]

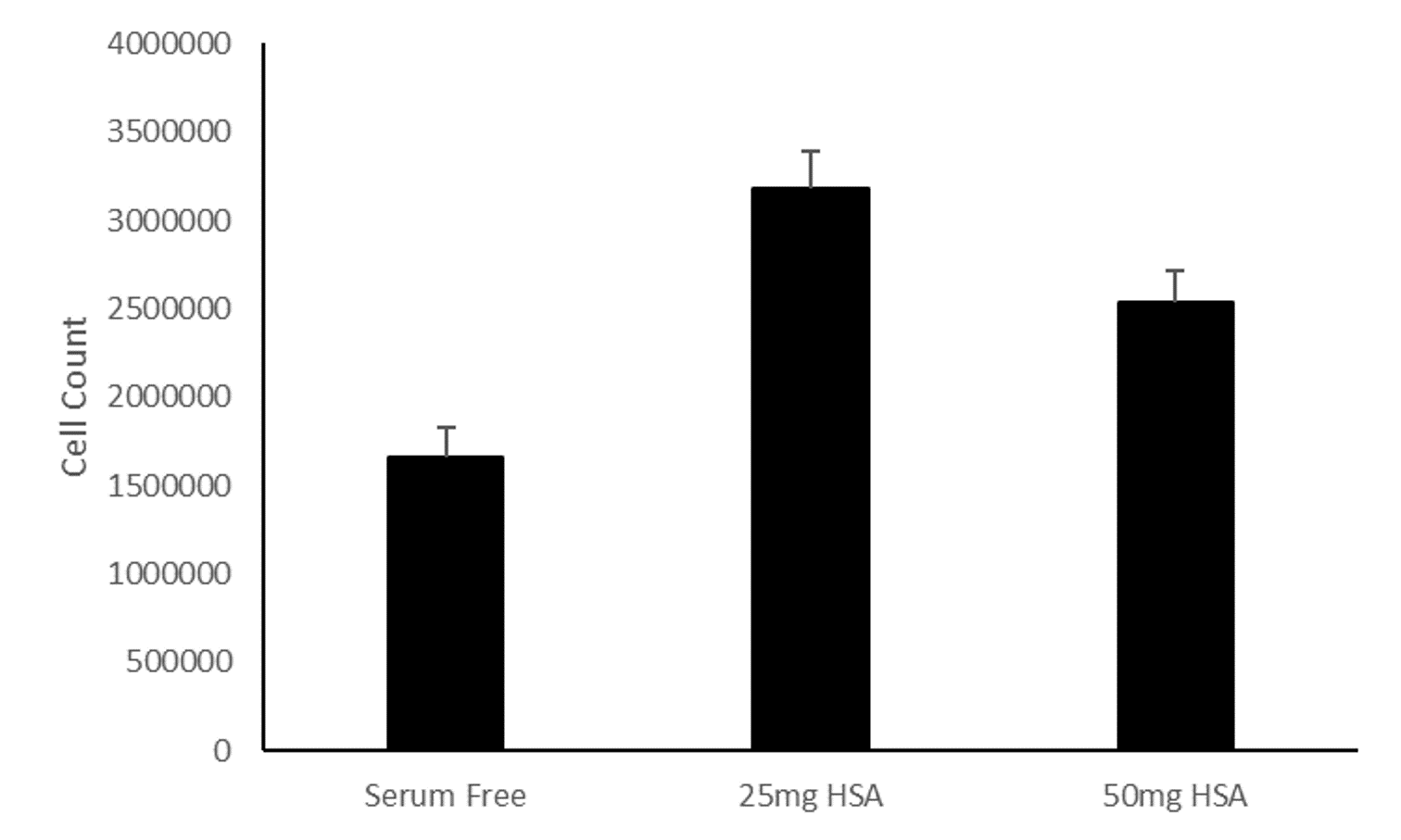

Supplement: Figure S3 — Increased cell counts is demonstrated in 25 mg/ml (p < 0.001) and 50 mg/ml (p < 0.001) albumin containing cultures compared to serum starved control. Values are mean ± SD (n = 4). [file peerj-08-8568-s003.png]

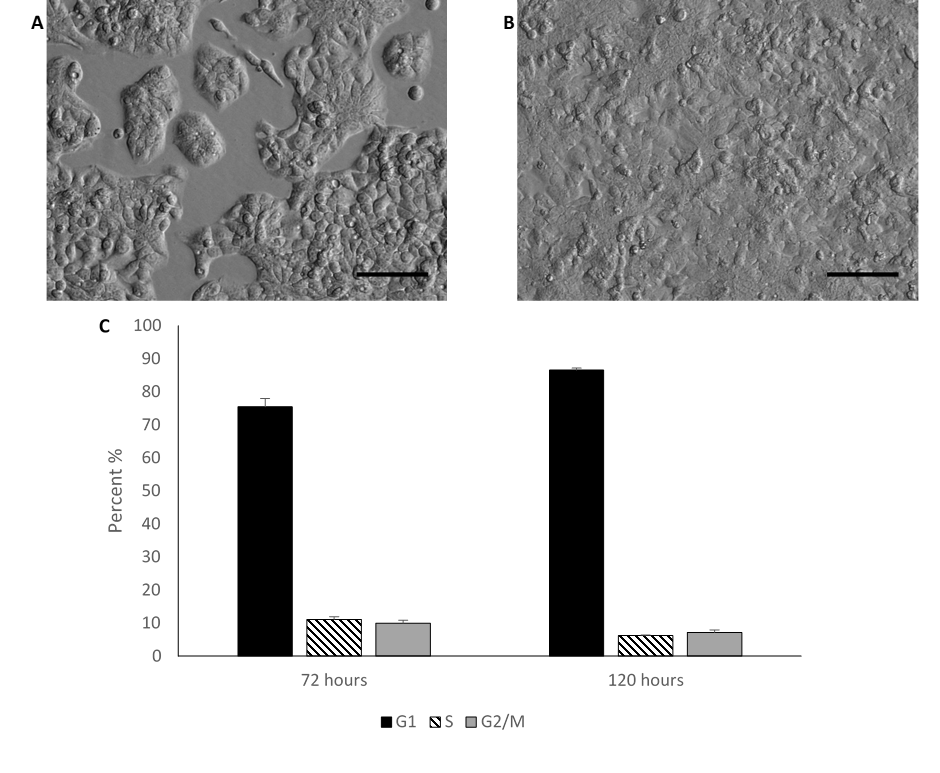

Supplement: Figure S4 — HEPG2/C3A cells grown in media supplemented with 10% foetal bovine serum (FBS) characteristically display a regular polygonal morphology and grow in monolayer colonies (A) after 72 h in culture, (B) after 120 h in culture (confluent). (C) Bar chart demonstrating the cell cycle stages calculated using the Watson pragmatic algorithm. Values are mean ± SD (n = 2). [file peerj-08-8568-s004.png]

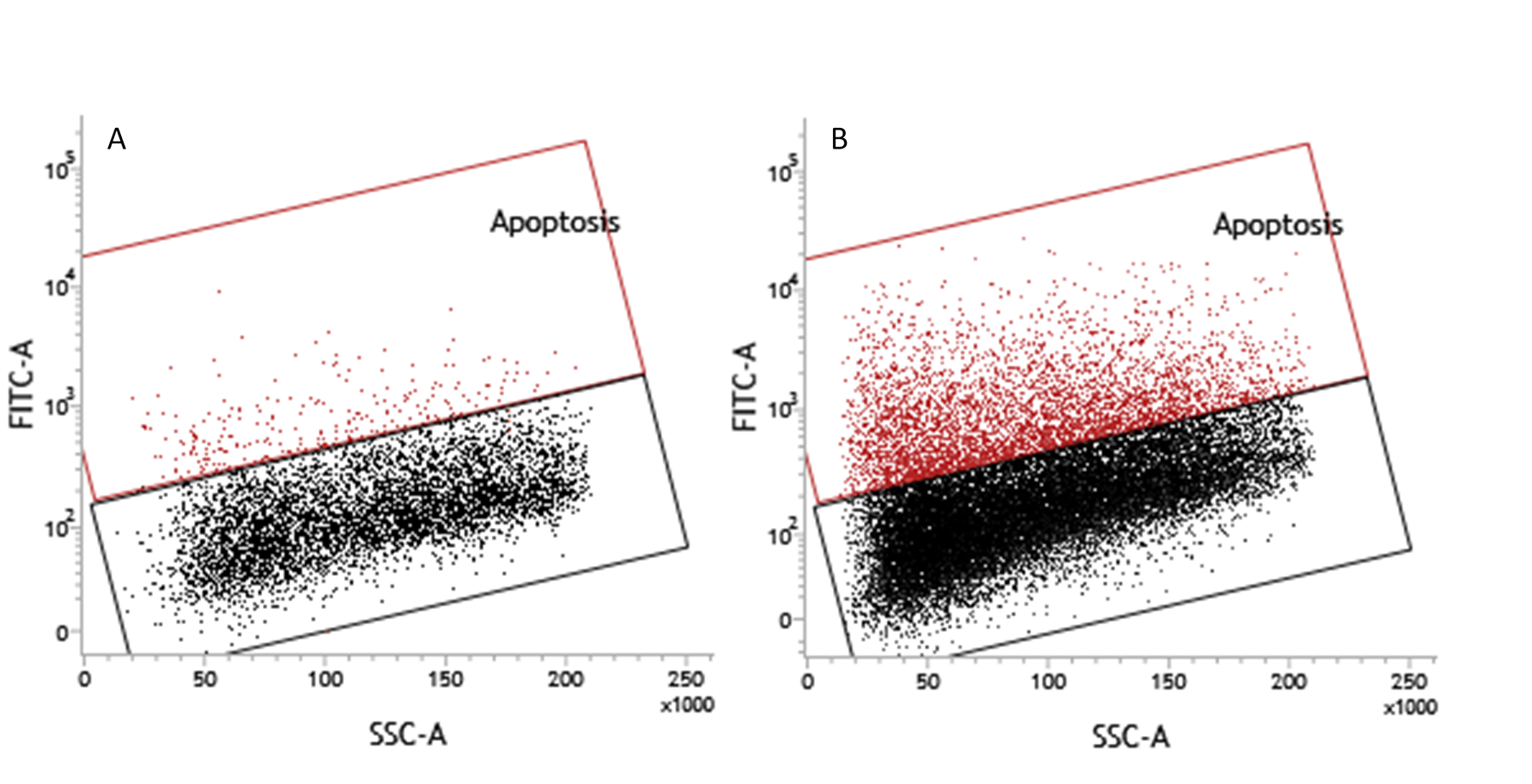

Supplement: Figure S5 — TUNEL assay of HEPG2/C3A cells demonstrates apoptosis at 4% and 16.6% after (A) 48 and (B) 72 h (image from Fig. 3 a of main text) of serum starvation respectively. [file peerj-08-8568-s005.png]

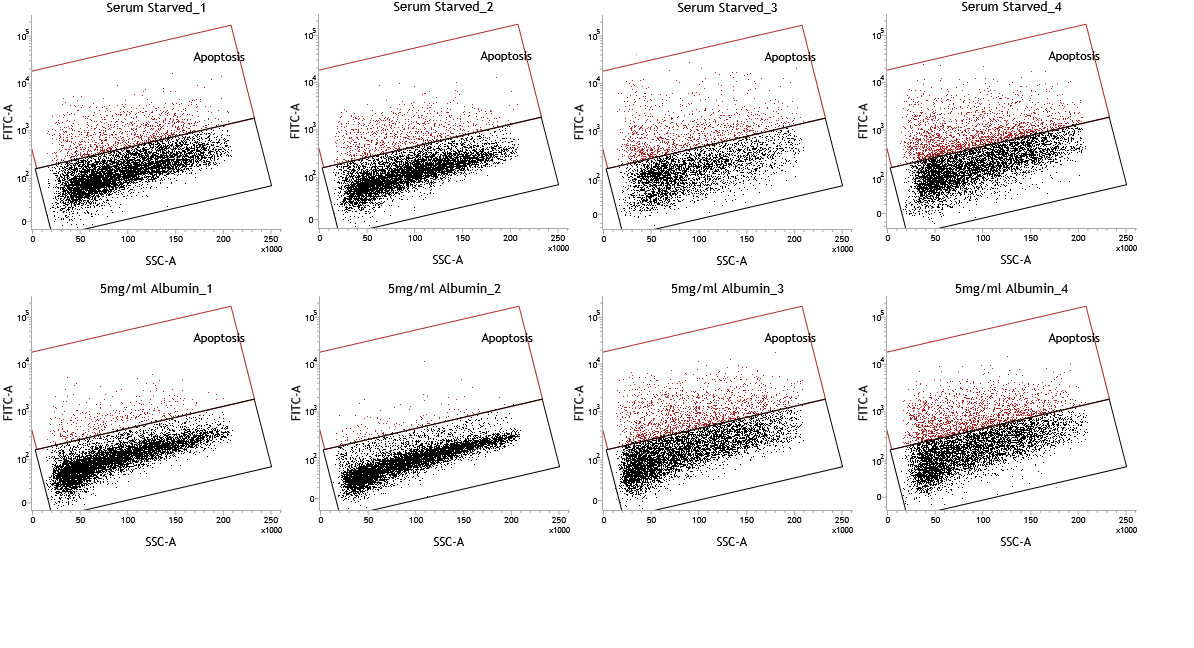

Supplement: Supplemental Information 6 — Individual dot plots of the TUNEL assay that were presented as overlays in Fig. 3. [file peerj-08-8568-s006.png]

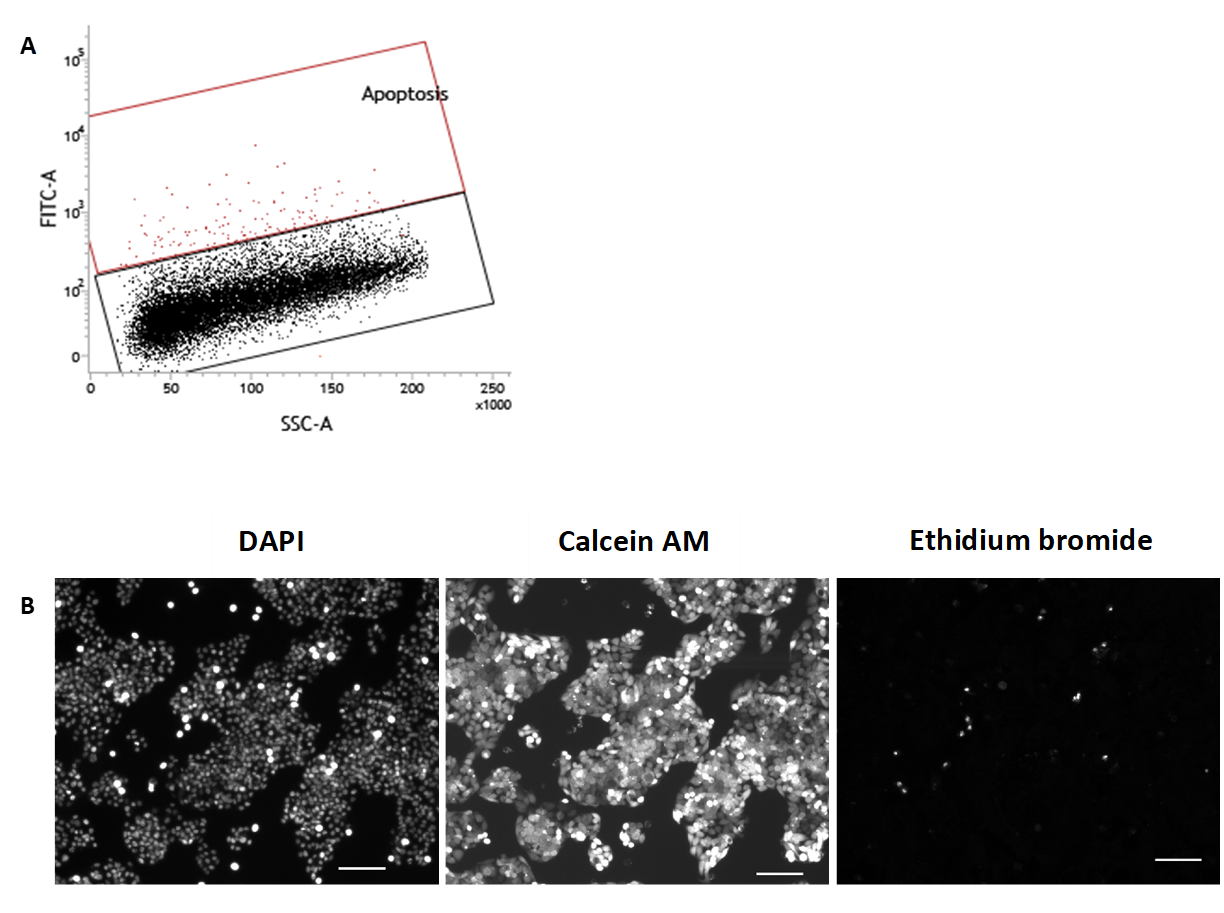

Supplement: Figure S7 — HepG2/C3A cells cultured in media containing 10% FBS for 72 h were analysed by (A) TUNEL assay using a flow cytometer for apoptosis (0.8 ± 0.2 %) (n = 2). (B) fluorescence microscopy demonstrates 1) DAPI (nuclear), 2) calcein AM (cytoplasmic) and 3) ethidium bromide (nuclear) staining. Scale bar = 100 μm. A necrotic index of 1.7 ± 0.8% was calculated as the percentage of necrotic cells (ethidium bromide) from the total cell count (DAPI) (n = 2). [file peerj-08-8568-s007.png]

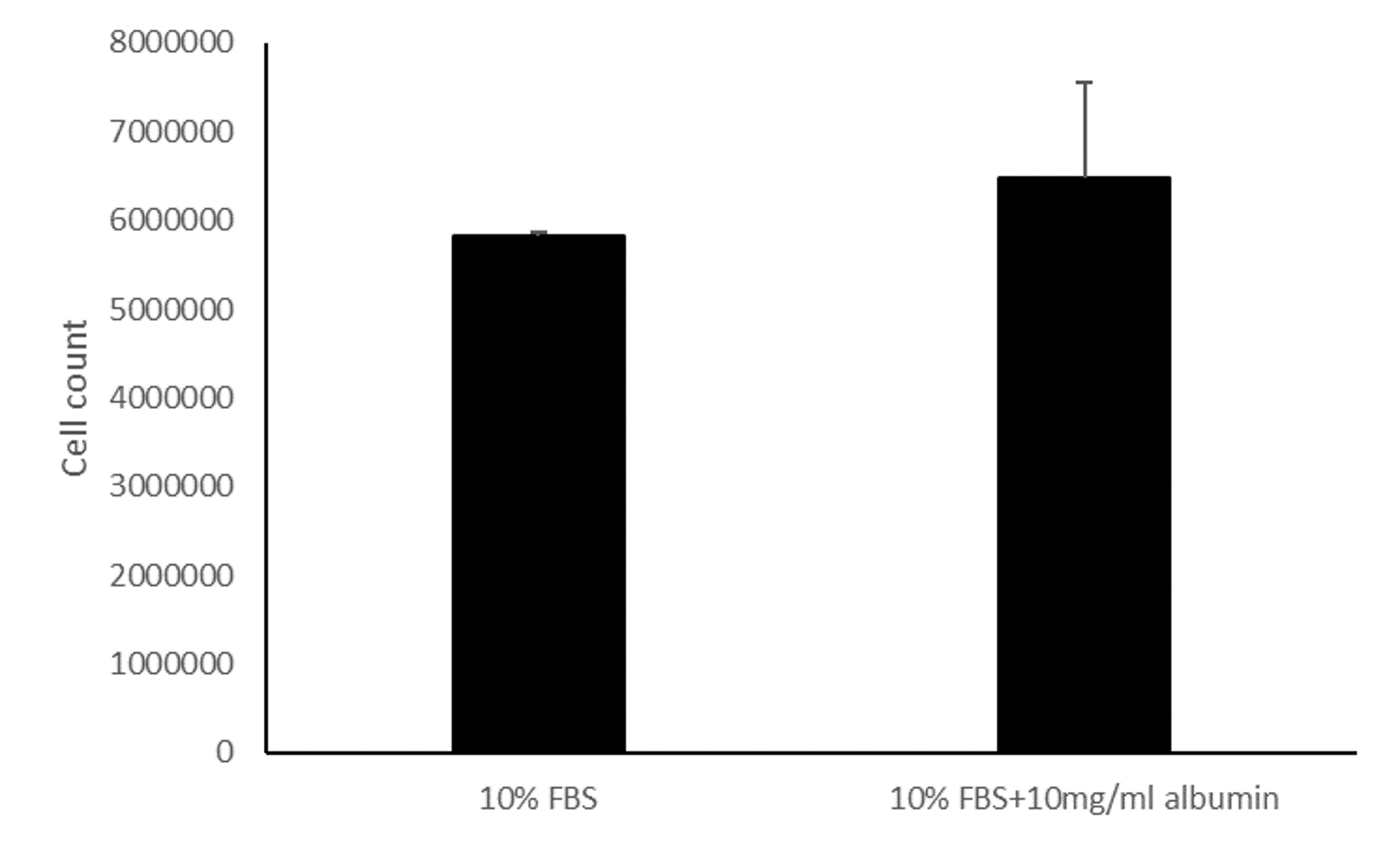

Supplement: Figure S8 — Mean cell counts of HepG2/C3A cells cultured for 72 h in media containing 10% FBS (5830000 ± 40000) and media containing 10% FBS treated with 10mg/ml albumin (6470000 ± 1083097). Values are mean ± SD (n = 3). [file peerj-08-8568-s008.png]

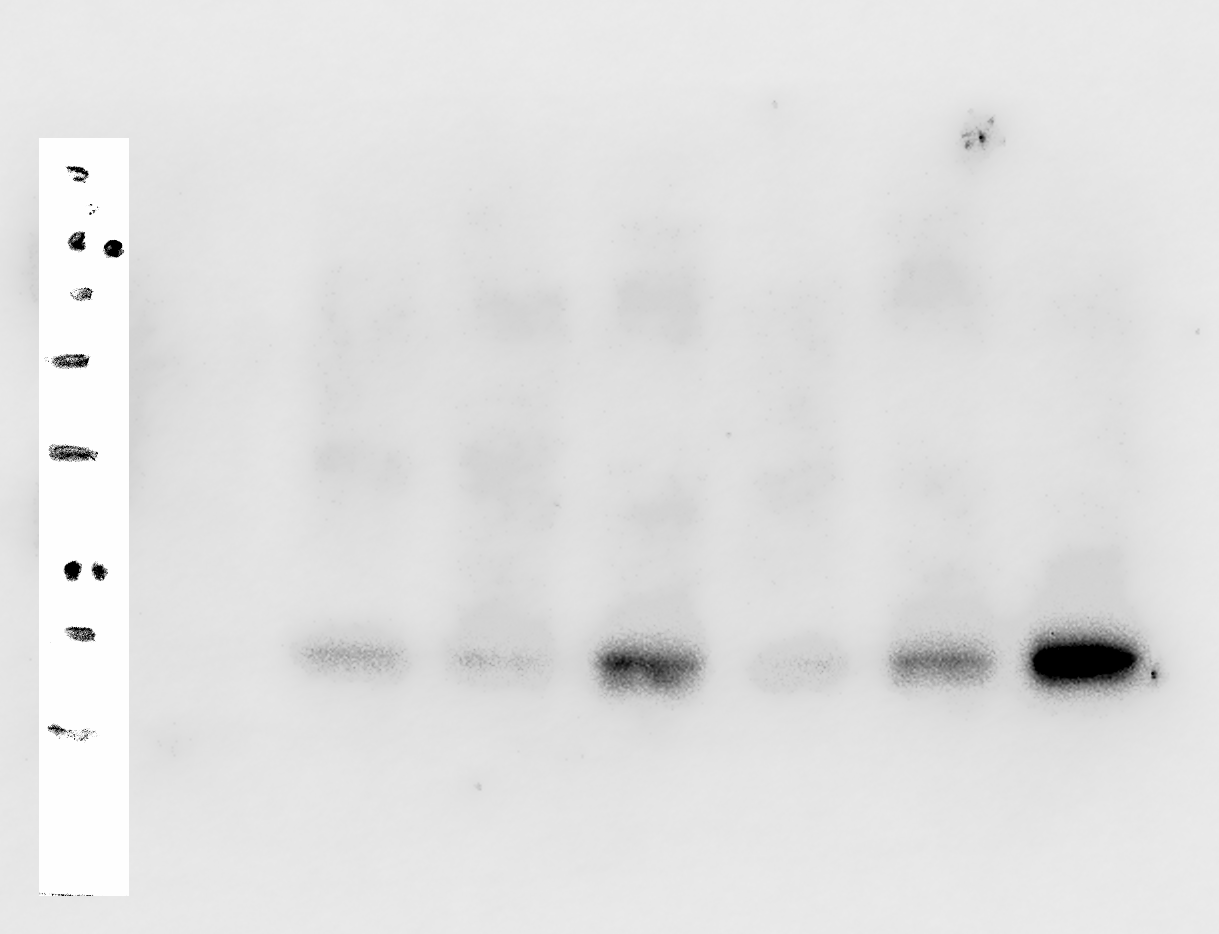

Supplement: Supplemental Information 2 — P21 western blot lanes 1–8 respectively: (1) Ladder, (2) mixed lysate (proliferating cells), (3) 5 mg/ml HSA (a), (4) 50 mg/ml HSA (a), 5) Serum starved (a), (6) 5 mg/ml HSA (b), (7) 50 mg/ml HSA (b), (8) Serum starved (b). This blot contains biological replicates a and b of serum starved, 5 mg/ml albumin and 50 mg/ml albumin treated cultures. 50 mg/ml albumin sample b is also included in p21 western blot 2 as the common sample for normalisation. [file peerj-08-8568-s010.png]

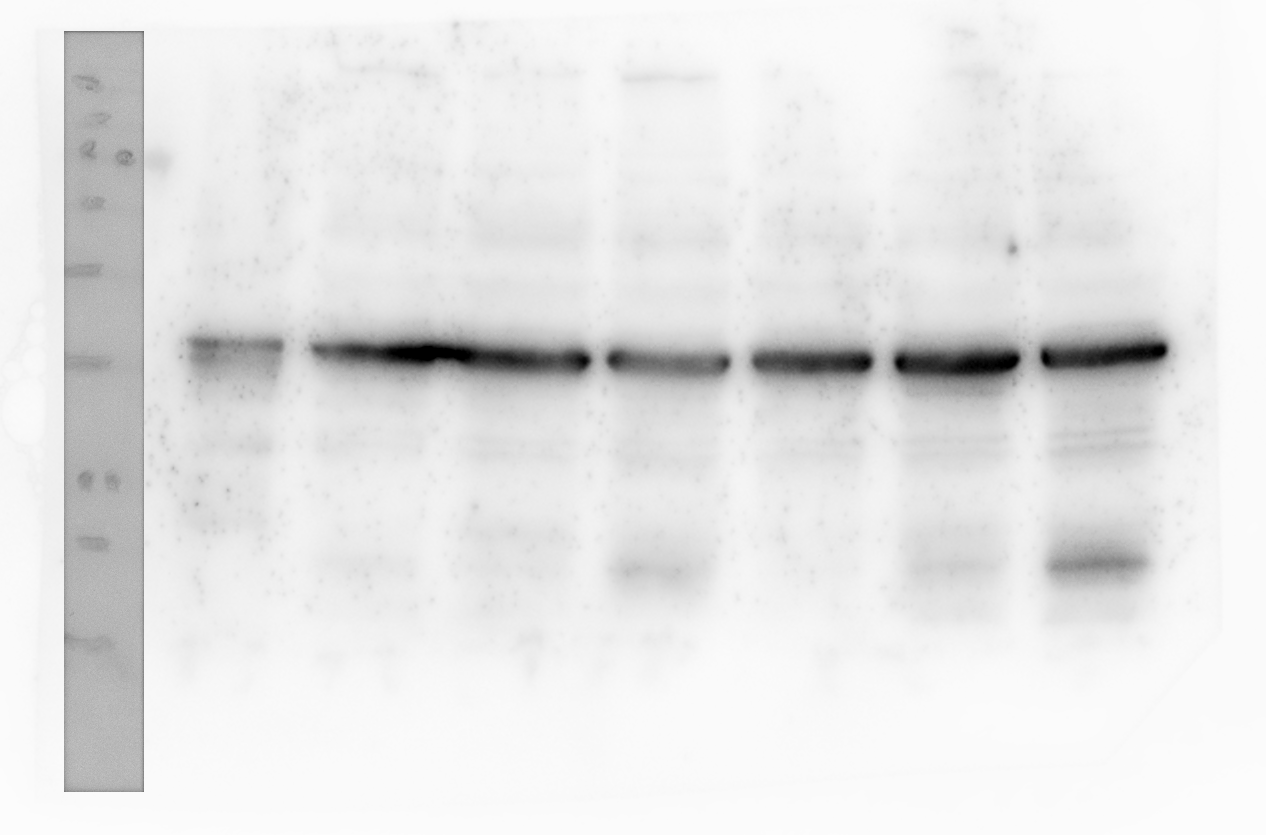

Supplement: Supplemental Information 3 — GAPDH western blot control for p21 western blot 1 lanes 1–8 respectively: (1) Ladder, (2) mixed lysate (proliferating cells), (3) 5 mg/ml HSA (a), (4) 50 mg/ml HSA (a), (5) Serum starved (a), (6) 5 mg/ml HSA (b), (7) 50 mg/ml HSA (b), (8) Serum starved (b). This blot contains biological replicates a and b of serum starved, 5 mg/ml albumin and 50 mg/ml albumin treated cultures. [file peerj-08-8568-s011.png]

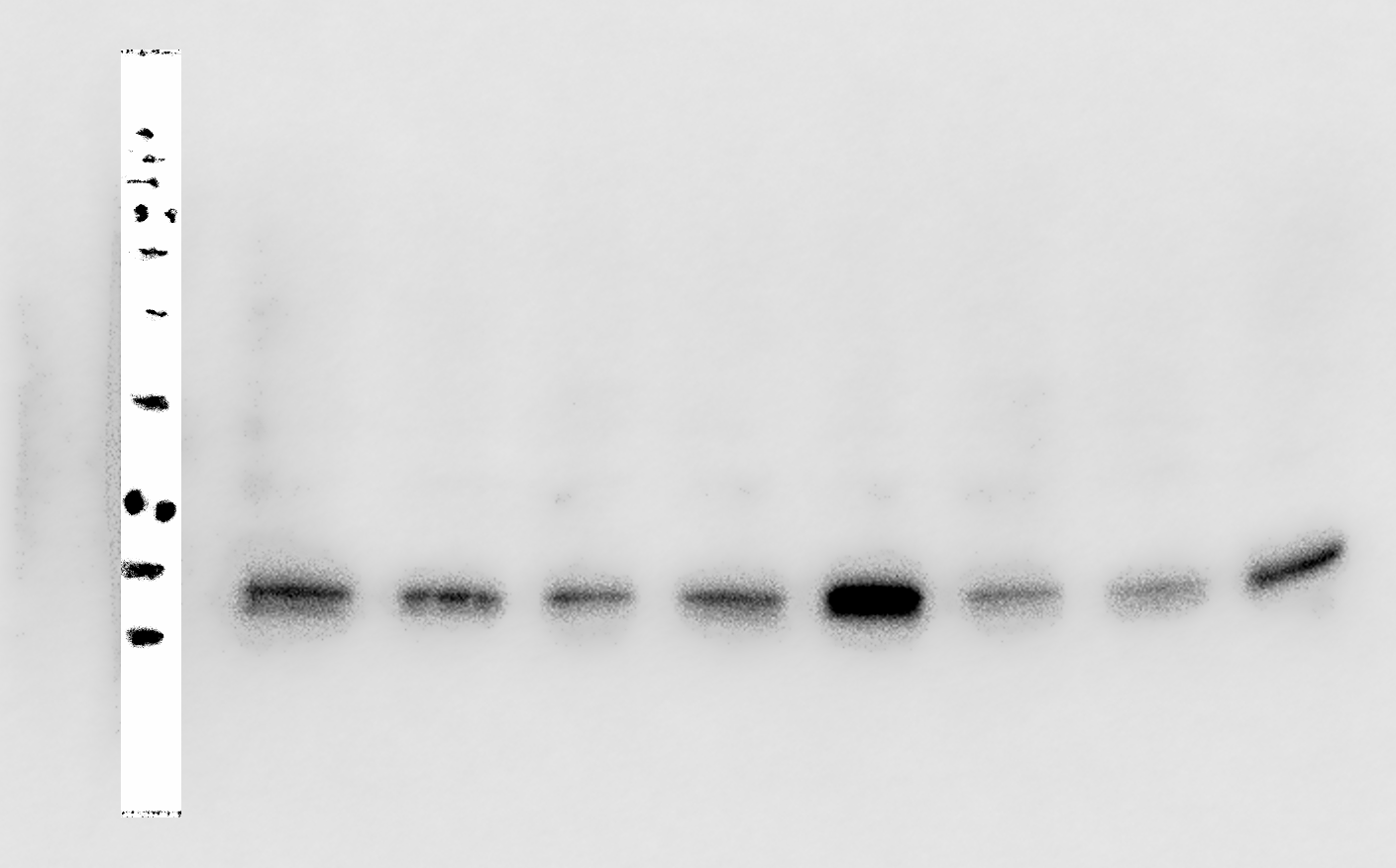

Supplement: Supplemental Information 4 — P21 western blot lanes 1–9 respectively: (1) Ladder, (2) mixed lysate, (3) mixed lysate, (4) 50 mg/ml HSA (b), (6) 5 mg/ml HSA (c), (6) Serum starved (c), (7) 50 mg/ml HSA (c), (8) 5 mg/ml HSA (d), (9) Serum starved (d). This blot contains biological replicates c and d of serum starved and 5 mg/ml albumin treated cultures. It also includes 50 mg/ml albumin treated culture samples b and c. 50 mg/ml albumin sample b is also included in p21 western blot 1 as the common sample for normalisation. [file peerj-08-8568-s012.png]

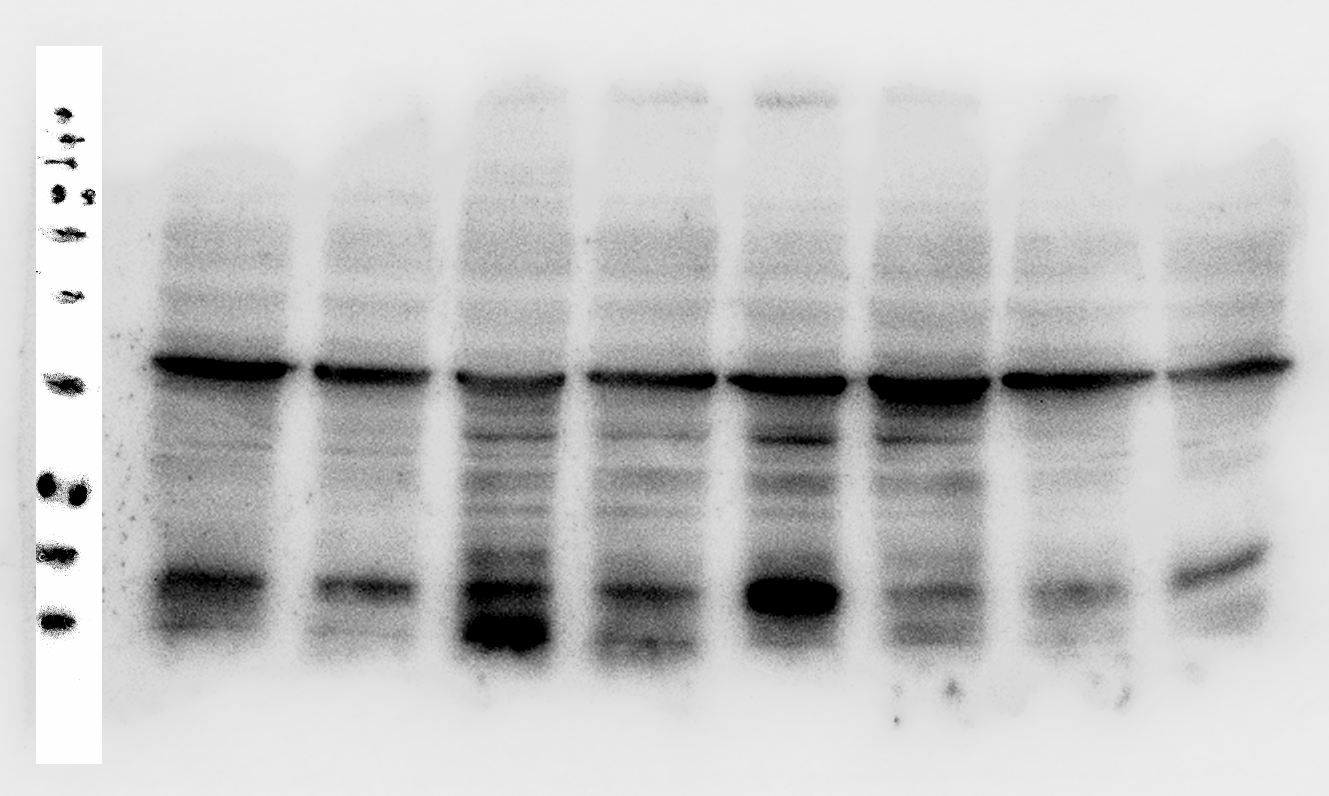

Supplement: Supplemental Information 5 — GAPDH western blot control for p21 western blot 2 lanes 1–9 respectively: (1) Ladder, (2) mixed lysate, (3) mixed lysate, (4) 50 mg/ml HSA (b), (6) 5 mg/ml HSA (c), (6) Serum starved (c), (7) 50 mg/ml HSA (c), (8) 5 mg/ml HSA (d), (9) Serum starved (d). This blot contains biological replicates c and d of serum starved and 5 mg/ml albumin treated cultures. It also includes 50 mg/ml albumin treated culture samples b and c. 50 mg/ml albumin sample b is also included in p21 western blot 1 as the common sample for normalisation. [file peerj-08-8568-s013.png]

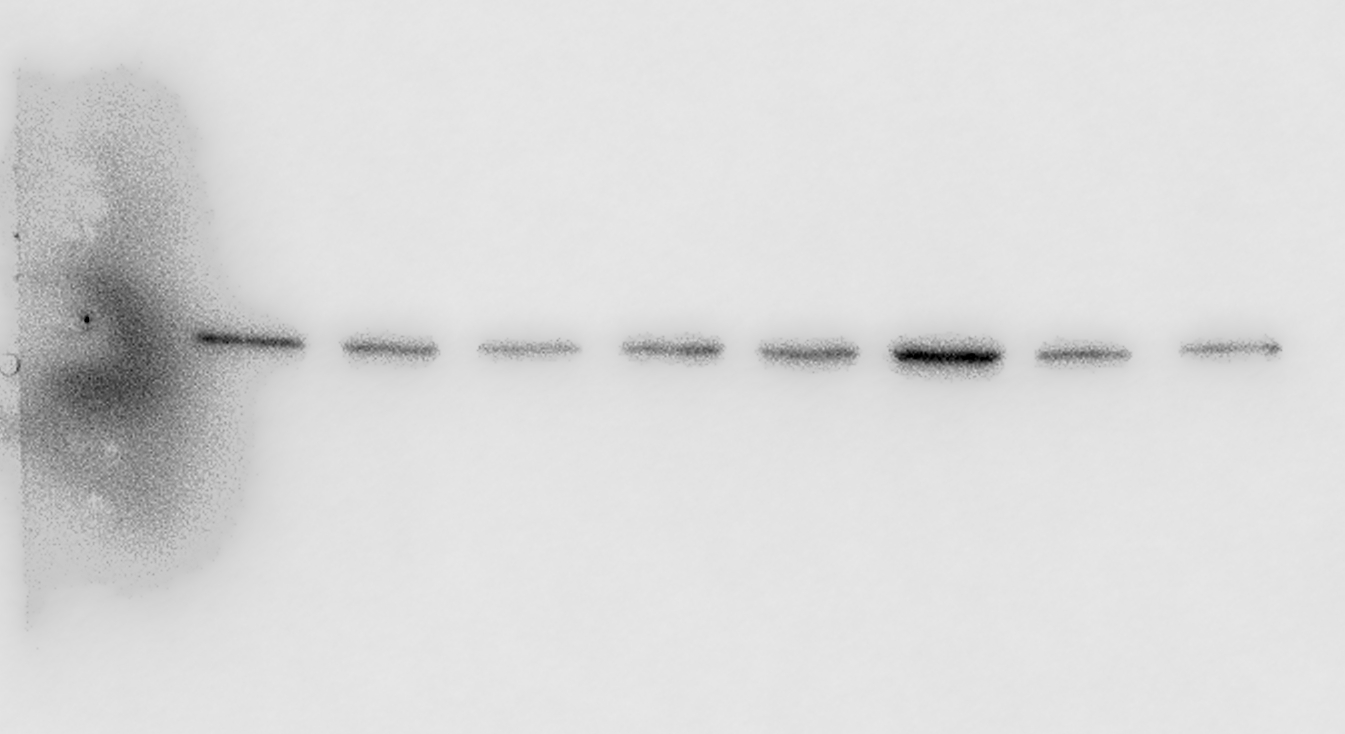

Supplement: Supplemental Information 6 — Cyclin D1 western blot lanes 1–9 respectively. (1) Ladder, (2) 5 mg/ml HSA (a), (3) 50 mg/ml HSA (b), (4) Serum starved (a), 5) 5 mg/ml HSA (b), 6) 50 mg/ml HSA (c), (7) Serum starved (b), 8) 5 mg/ml HSA (c), 9) Serum starved (c) . This blot contains biological replicates a, b and c of serum starved and 5 mg/ml albumin; and replicates b and c of 50 mg/ml albumin treated cultures. [file peerj-08-8568-s014.png]

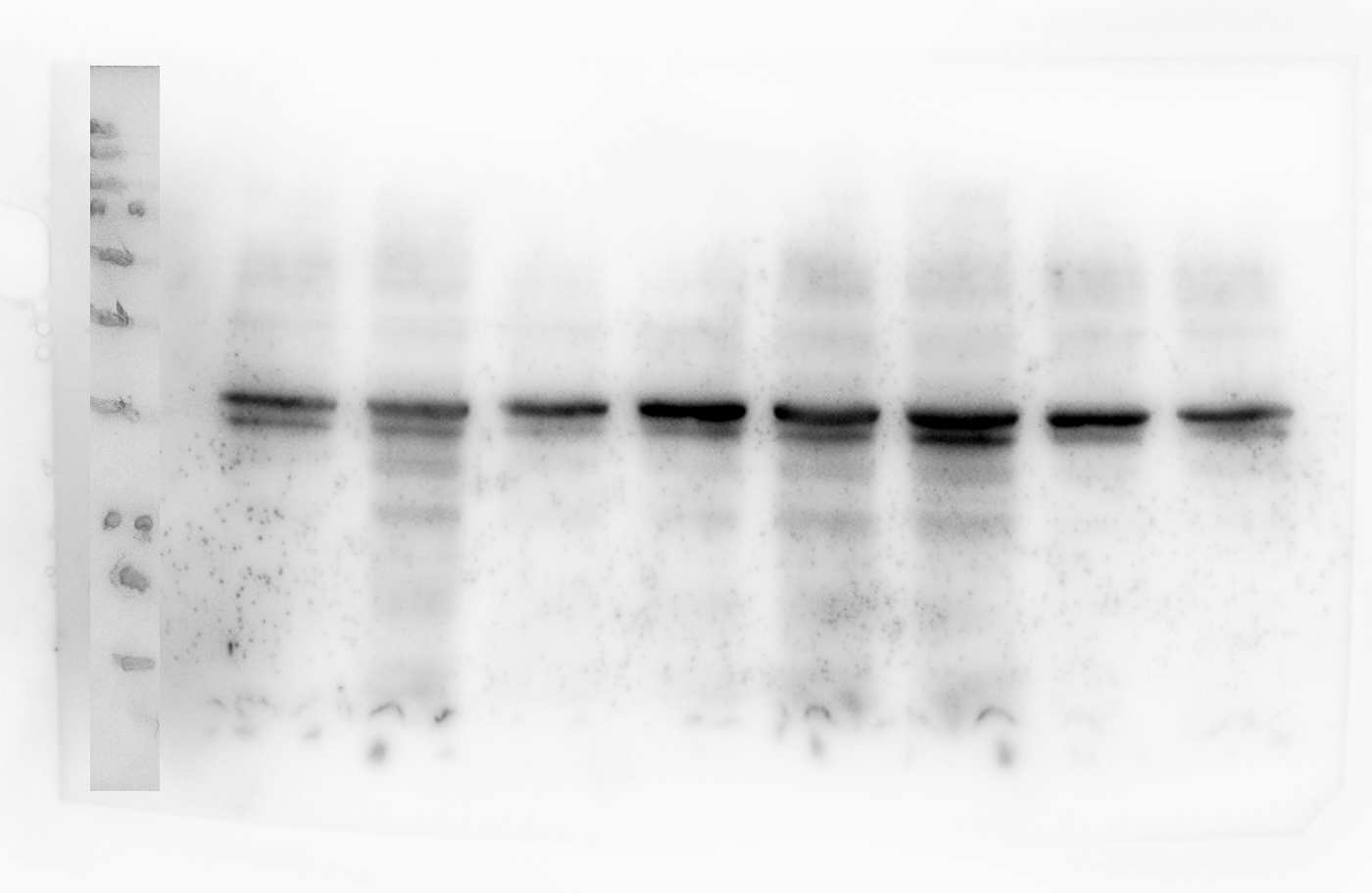

Supplement: Supplemental Information 7 — Cyclin D1 western blot lanes 1–9 respectively. (1) Ladder, (2) 5 mg/ml HSA (a), (3) 50 mg/ml HSA (b), (4) Serum starved (a), (5) 5 mg/ml HSA (b), (6) 50 mg/ml HSA (c), (7) Serum starved (b), 8) 5 mg/ml HSA (c), 9) Serum starved (c) . This blot contains biological replicates a, b and c of serum starved and 5 mg/ml albumin; and replicates b and c of 50 mg/ml albumin treated cultures. [file peerj-08-8568-s015.png]

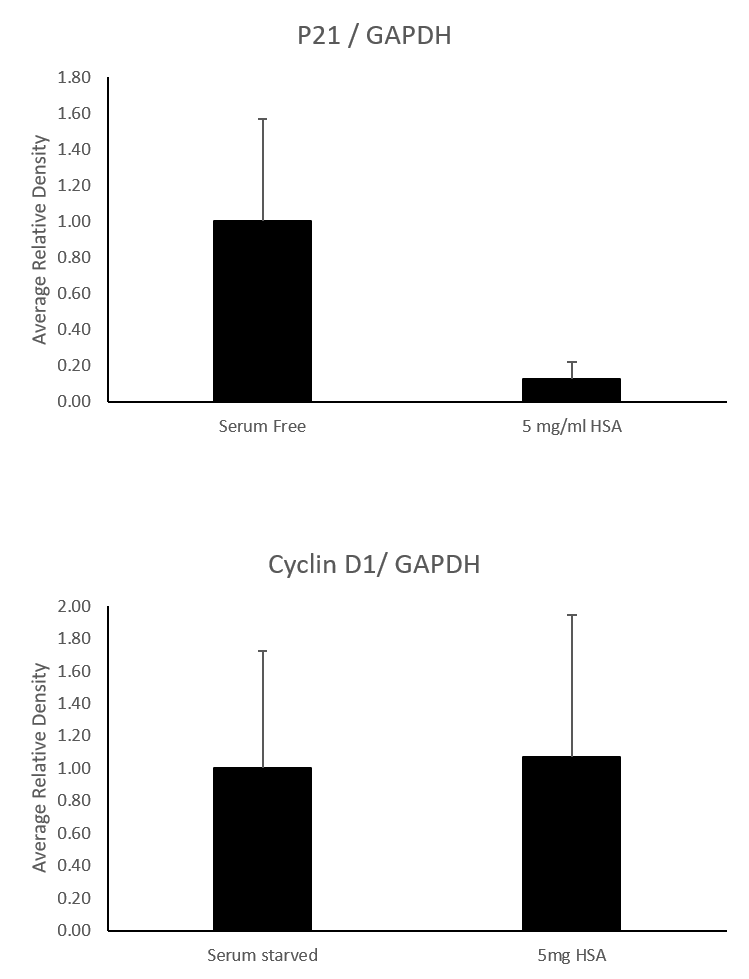

Supplement: Supplemental Information 8 [file peerj-08-8568-s016.png]
